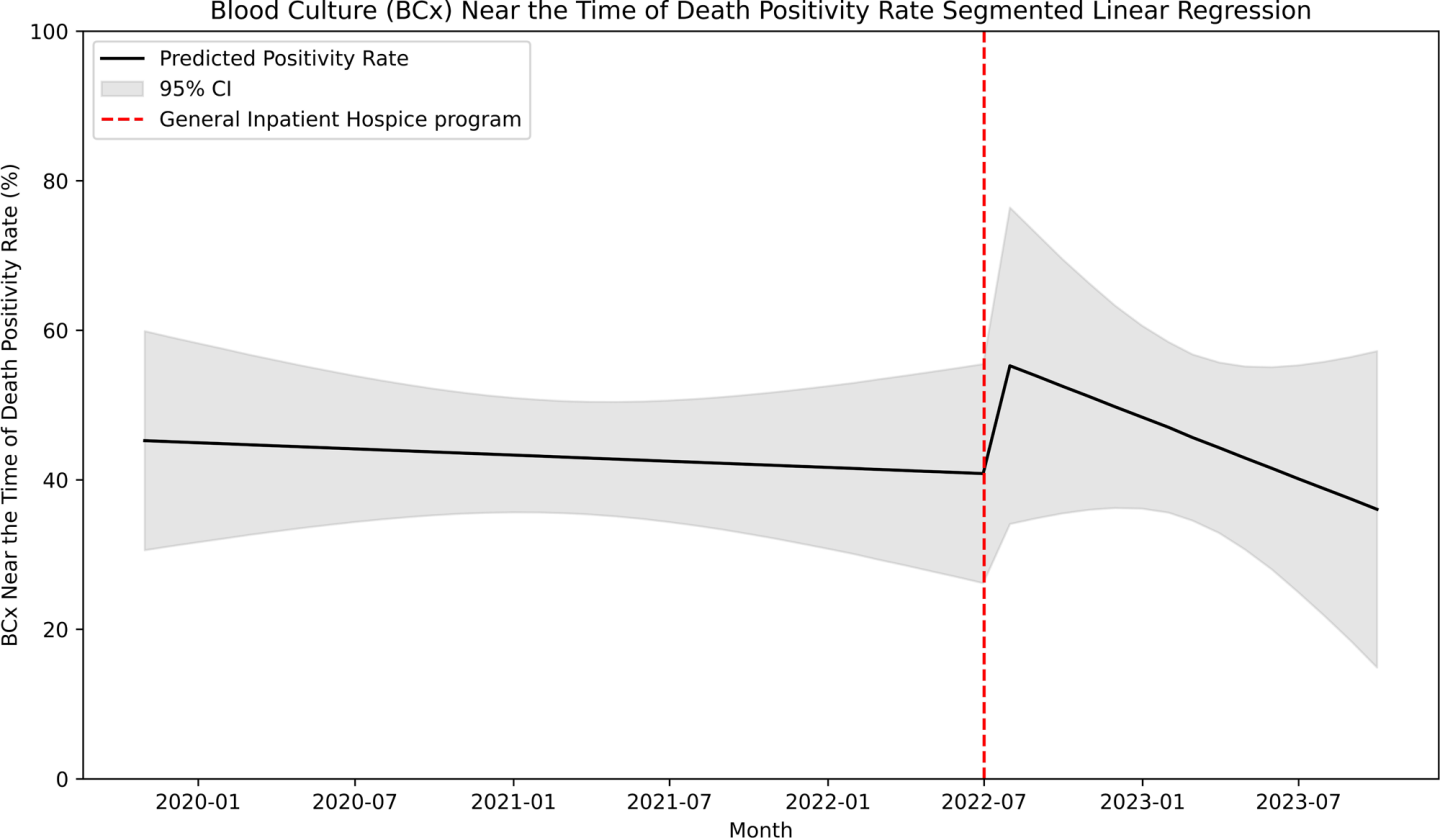# Inpatient Hospice Impact on Blood Culture Practices Near the Time of Death, Tertiary Center, Northern California, 2019–2023

**DOI:** 10.1017/ash.2024.222

**Published:** 2024-09-16

**Authors:** Guillermo Rodriguez Nava, Sulwan AlGain, Karen McIntyre, Eugenia Miranti, John Shepard, Lisa Shieh, Mindy Sampson, Jorge Salinas

**Affiliations:** Stanford University School of Medicine; Stanford University; King Faisal Specialist Hospital and Research Center;; Stanford Healthcare

## Abstract

**Introduction:** Many central line-associated bloodstream infections are identified in patients nearing the end of life. Stanford Health Care recently introduced the General Inpatient Hospice program. This program offers inpatient hospice care for patients who, due to uncontrolled symptoms, cannot be discharged to a hospice facility or receive home hospice care. We investigated whether this program would impact blood cultures practices near the time of death. **Methods:** We performed a retrospective cohort study at Stanford Health Care using records of blood culture events from May 2019 to October 2023. We defined a blood culture near-death as those collected within 2 days before the date of death. We performed an interrupted time series linear regression before and after the implementation of the General Inpatient Hospice program on July 1, 2022 to assess blood culture intensity near-death. Blood culture intensity was defined as the proportion of cultures collected near-death in relation to the total number of blood cultures. Additionally, we calculated blood culture positivity rate, which was defined as the proportion of positive blood cultures among all those collected during our study period. **Results:** Out of 220,269 blood cultures from 24,955 unique patients, a total of 6,147 cultures (9%) were obtained near the time of death. Among these subjects, the median age was 65 years (range 20–102), with 43% identifying as being of White race-ethnicity and 57% as male. Of these cultures, 3044 were positive (49.5%), with Escherichia coli (618, 24%), Klebsiella pneumoniae (341, 13%), and Staphylococcus aureus (166, 10%) being the most common organisms. After the implementation of the General Inpatient Hospice program, the median enrollment was 12 patients (range 3–18) and the median mortality rate was 2.3% (range 2–3%). The blood culture intensity near death decreased by 0.81%, a change that was not statistically significant (95% CI -2.4% to 0.8%, p=.32; Figure 1). Subsequently, the blood culture intensity showed a non-significant increasing trend of 0.05% (95% CI -0.1% to 0.2%, p=0.53). The blood culture positivity rate near the time of death increased by 16% following the intervention, but this increase was not statistically significant (95% CI – 11.8% to 43.3%, p=.26; Figure 2), and it was followed by a non-significant downtrend of 1.9% (95% CI -3.9% to 1.4%, p=.36). **Conclusion:** We found no significant association between the implementation of an inpatient hospice program and blood culture practices near the time of death, likely due to low patient enrollment.

**Figure:**
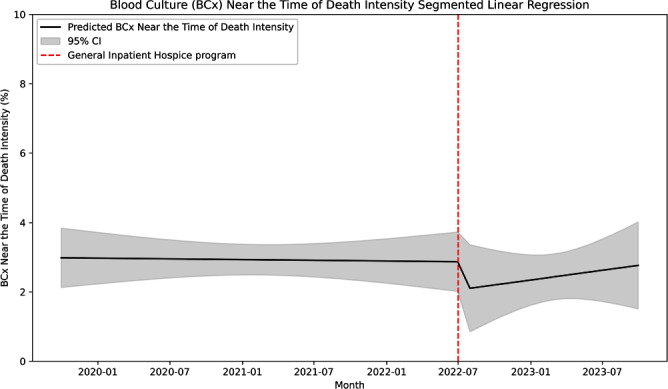

**Figure:**